# Pesticide Residues in Apples and Pears: A Deterministic Assessment of Chronic Exposure and Non-Carcinogenic Risk for European Consumers

**DOI:** 10.3390/molecules31050767

**Published:** 2026-02-25

**Authors:** Jarosław Chmielewski, Barbara Gworek, Magdalena Florek-Łuszczki, Jarogniew J. Łuszczki

**Affiliations:** 1 Department of Public Health, Academy of Medical Sciences of Applied and Holistic Sciences, 02-304 Warsaw, Poland; jaroslaw.chmielewski@amh.edu.pl; 2 Department of Environmental Chemistry and Risk Assessment, Institute of Environmental Protection-National Research Institute, 02-170 Warsaw, Poland; barbara.gworek@ios.edu.pl; 3 Department of Medical Anthropology, Institute of Rural Health, 20-090 Lublin, Poland; florek.magdalena@imw.lublin.pl; 4 Department of Occupational Medicine, Medical University, 20-090 Lublin, Poland

**Keywords:** pesticide residues, chronic exposure, risk assessment, health risk, apples, pears

## Abstract

(1) Pome fruits (apples and pears) are among the most frequently consumed fruits in Europe and may contribute to dietary exposure to pesticide residues. Although residue levels generally comply with maximum residue limits (MRLs), even low concentrations may cumulatively contribute to chronic health risks under conditions of frequent and long-term consumption. This study aimed to quantitatively assess dietary exposure and the potential non-carcinogenic health risks associated with pesticide residues in apples and pears, using representative monitoring and consumption data. (2) The assessment was based on results of the Polish national official monitoring program for pesticide residues in food, specifically apples and pears sampled in 2022, as reported by the National Institute of Public Health (NIZP-PZH). These data were combined with age- and body weight-specific consumption scenarios derived from FAO/WHO GEMS/Food cluster diets and national Polish statistics. For the most frequently detected pesticides (captan, flonicamid, acetamiprid and fosetyl-Al in apples; captan and acetamiprid in pears), the mean and 95th percentile concentrations were used to estimate the estimated daily intake (EDI). Non-carcinogenic risk was characterized using the hazard quotient (HQ = EDI/ADI) and the cumulative Hazard Index (HI). The hazard quotient (HQ) was calculated as the ratio of estimated daily intake to the acceptable daily intake (HQ = EDI/ADI), while the Hazard Index (HI) was defined as the sum of individual HQ values for pesticides detected in a given commodity and exposure scenario (HI = ΣHQ). Calculations were performed separately for children and adults under several dietary scenarios (Polish general population, German child, German general population, GEMS/Food G08). (3) For all pesticides and exposure scenarios, the HQ values were well below 1, indicating no exceedance of the acceptable daily intake (ADI). The highest chronic exposure was observed for apples in children (German child scenario), with the HQ values for captan, flonicamid and acetamiprid in the approximate range of 0.01–0.05, while the HI remained < 0.1 even under high-consumption conditions. In adults (Polish and German general populations, GEMS/Food G08), HQ values were approximately one order of magnitude lower than in children, and the cumulative HI values for both apples and pears were far below 1. The contribution of pears to total exposure was limited, reflecting lower consumption and fewer active substances detected. (4) This quantitative risk assessment, based on Polish monitoring data from 2022, indicates that under current residue levels and consumption patterns, chronic dietary exposure to pesticide residues from apples and pears does not pose a relevant non-carcinogenic health concern for either children or adults. Nevertheless, children consistently showed higher relative exposure than adults, underscoring the importance of age-stratified risk assessment and continued monitoring of residues in commonly consumed fruits. The findings support existing regulatory frameworks while justifying sustained, targeted surveillance of key active substances in pome fruits as part of public health prevention strategies.

## 1. Introduction

Pome fruits, particularly apples and pears, are among the most widely consumed fruits in Europe and contribute substantially to total fruit intake and diet quality. Europe is both a major producer and exporter of apples, with some countries consistently ranking among the leading suppliers on the global market [1,2]. In temperate regions, apples and pears are available year-round, and are consumed both fresh and as processed products (e.g., juices, purées, and concentrates), making them important vehicles of long-term dietary exposure to both beneficial nutrients and undesirable contaminants [1,2,3]. Within the European Union, Poland is one of the largest producers and exporters of apples, and pears are also an important, though smaller, pome fruit crop, which further increases the relevance of residue monitoring and risk assessment in this context [4,5].

From a nutritional standpoint, apples and pears are considered core components of healthy dietary patterns. They provide dietary fiber (including pectins), vitamin C, selected B-group vitamins, organic acids, and a wide range of phytochemicals with antioxidant and anti-inflammatory properties [6,7,8]. Owing to their low energy density and favorable nutrient profile, pome fruits are commonly recommended within fruit and vegetable intake targets. The World Health Organization (WHO) and many national dietary guidelines advise a minimum daily intake of 400–600 g of fruits and vegetables, typically corresponding to at least five servings per day, with fruits representing a considerable share of this recommendation [3,7,9]. In practice, apples and pears often account for a substantial proportion of fruit intake, especially in children, older adults, and populations living in temperate-climate countries [2,3,9]. In Poland, household budget surveys and agricultural statistics confirm the central role of apples in fruit consumption, whereas pears, despite their lower intake, remain a relevant contributor to overall pome fruit exposure [4,10].

The highest apple consumption over the years of 2022/2023 was recorded in China (53% of global consumption), the European Union (14%), Turkey (6%), the United States (5%), India (3%), Russia (3%), Iran (2%), Brazil (2%), Ukraine (2%), Mexico (1%), and other countries (10%) [5].

The area of cultivation and the production of apples and pears in Poland over the years 2020–2024 are presented in Table 1, while Table 2 presents the export of apples and pears in the same period [10].

However, the intensive production systems needed to maintain high yields, visual quality, and storability of pome fruits rely heavily on plant protection products (PPPs), including fungicides and insecticides. In commercial orchards, 20–30 pesticide applications per season are not uncommon, and a large proportion of the applied active substances may reach the fruit surface [8,11]. Although modern PPPs are subject to strict authorization procedures, their use inevitably leads to the presence of pesticide residues in food of plant origin. Food is therefore a major route of human exposure to pesticide residues in the general population [12,13]. Chronic exposure to pesticides has been associated in epidemiological and toxicological studies with a range of adverse health outcomes, including neurodevelopmental effects, endocrine disruption, and metabolic and reproductive disorders, particularly in susceptible subgroups such as children and pregnant women [14,15,16,17].

Pesticide residues detected in routine monitoring are generally within the maximum residue limits (MRLs) set by regulatory authorities, and only a small proportion of samples exceed these legal thresholds [18,19,20]. Nonetheless, compliance with MRLs does not automatically imply the absence of health risk, as MRLs are enforcement tools rather than toxicologically based safety limits. Health-based guidance values, such as the acceptable daily intake (ADI) and acute reference dose (ARfD), are derived from toxicological studies and are used in risk assessment to characterize chronic and acute dietary exposure [15,16]. Even low residue levels, when combined with frequent and long-term consumption of commonly eaten fruits such as apples and pears, may cumulatively contribute to non-cancer health risks, particularly in sensitive groups such as children [11,15,16,17,18].

For these reasons, many countries have implemented national monitoring and official control programs for pesticide residues in food, aligned with European Union (EU) and international requirements [18,19,20]. In Poland, such monitoring is coordinated by the Chief Sanitary Inspectorate and scientifically evaluated by the National Institute of Public Health—National Institute of Hygiene—National Research Institute (NIZP PZH-PIB), which regularly publishes detailed reports on pesticide residues in foods available on the Polish market [21,22]. These programs generate large datasets on the occurrence and levels of pesticide residues in different food categories. When combined with harmonized consumption data (e.g., FAO/WHO GEMS/Food cluster diets or national dietary surveys), these monitoring results provide the basis for quantitative dietary exposure assessment [15,16,19]. In such assessments, estimated daily intake (EDI) values are calculated and compared with the ADI to derive the hazard quotient (HQ), while the cumulative Hazard Index (HI), defined as the sum of HQ values for multiple compounds, is used to characterize the potential non-carcinogenic risk associated with mixtures [16,17,18]. This approach is consistent with current risk assessment practices recommended by international bodies such as EFSA and FAO/WHO.

Despite the central role of apples and pears in European diets, detailed commodity-specific assessments of dietary exposure to pesticide residues in these fruits remain relatively limited compared with other food groups. This is particularly relevant in high-production countries such as Poland, where domestic consumption coexists with extensive international trade in fresh and processed pome fruits [4,5,10,21].

The scope of official control in Poland primarily covers food safety (identified by the health quality of food) and its commercial quality and results directly from legal regulations in force in the EU [20]. Official food control is carried out based on EU and national control programs. Annually updated implementing regulations (in the case of the 2022 studies, they were carried out in accordance with Commission Implementing Regulation (EU) No 2021/601 of 13 April 2021) specify food products and pesticides that should be monitored by all Member States under a multiannual, coordinated EU program (EU-coordinated control program, EUCP). Additionally, all Member States define the scope of national control (national control program, NP), taking into account elements such as the toxicity of pesticides approved for use, the results of previous control programs, the specific nature of agriculture in a given country, the share of product consumption in the national nutrition system, etc. [22].

The results of monitoring and official control of food contamination with pesticides available on the Polish market based on the analysis prepared by the NIZP PZH-PIB are shown in Table 3 and Table 4 [22].

Moreover, temporal trends in the national monitoring data indicate that multiple residues per sample are increasingly common, underscoring the importance of mixture-oriented indicators such as the HI [19,20,21].

In this context, the present study aims to perform a quantitative dietary exposure and non-carcinogenic risk assessment for selected pesticide residues in apples and pears placed on the Polish market. Using recent results from the Polish official monitoring program (2022), combined with age- and body weight-specific consumption scenarios derived from FAO/WHO GEMS/Food cluster diets and national statistics, we focus on the most frequently detected active substances—captan, flonicamid, acetamiprid, and fosetyl-Al in apples, and captan and acetamiprid in pears. EDI, HQ, and HI values are calculated for both children and adults under several dietary scenarios. By integrating Polish monitoring and consumption data in a framework consistent with international guidance, this work seeks to determine whether current residue levels and consumption patterns in pome fruits are compatible with public health protection goals and to identify potential priorities for continued surveillance and risk-based risk management.

## 2. Results

### 2.1. Occurrence of Pesticide Residues in Apples and Pears

The national monitoring data analyzed in this study confirm that pesticide residues are frequently detected in pome fruits available on the Polish market, but generally at levels compliant with the applicable maximum residue limits (MRLs). In the overall food monitoring system, 48.6% of all samples analyzed in 2022 contained residues of at least one pesticide, while 37.4% contained residues of two or more active substances (Table 3). Only 2.9% of samples showed exceedances of MRLs, indicating a relatively low rate of non-compliance in relation to the total number of samples tested [22].

With regard to pome fruits, 89 apple samples and 72 pear samples were analyzed under the official control program (Table 4). In 2022, a total of 89 apple samples were tested for the presence of 481 pesticides (including 87 samples taken from the market and two samples taken as part of border inspections). Of the apple samples, 76 were domestically produced, nine samples came from other Member States, three from third countries, and for one sample, the country of origin was not identified. Residues of a total of 36 pesticides were found in all tested samples, and the number of positive results (i.e., ≥LOQ) was 261. Seven samples exceeded one MRL, but after taking into account the default expanded uncertainty of 50%, one result was considered non-compliant with the MRL. No residues of any of the tested compounds were detected in 16 samples (18%). Pesticide residues were detected in 73 samples (82%), including 67 samples (75%) containing at least two pesticides. None of the samples contained more than eight pesticides. The most frequently detected pesticides in apples were: captan (in 60 samples; 67.4%), flonicamid (in 30 samples; 33.7%), acetamiprid (in 26 samples; 29.2%), and fosetyl-Al (in 26 samples; 29.2%). Furthermore, the presence of fludioxonil in 13 samples (14.6%), tebuconazole in 11 samples (12.4%), boscalid in 10 samples (11.2%), and cyprodinil in nine samples (10.1%) should be noted. In 2022, 72 samples of pears collected from the market were tested for the presence of 479 pesticides. Of the samples, 62 came from Poland, seven from other Member States, and three from third countries. All of the samples tested contained residues of a total of 29 pesticides, and the number of positive results (i.e., ≥LOQ) was 159. One sample exceeded one MRL. After taking into account the default expanded uncertainty of 50%, this result was considered non-compliant with the MRL. Only 10 of the samples (14%) did not contain residues of any of the tested compounds. Pesticide residues were detected in 62 samples (86%), including 43 samples (60%) containing at least two pesticides. None of the samples contained more than nine pesticides. The most frequently detected pesticides in pears were: captan (in 42 samples; 58.3%) and acetamiprid (in 18 samples; 25.0%). It should also be noted that boscalid was present in 11 samples (15.3%), fludioxonil in 11 samples (15.3%), chlorantraniliprole in 10 samples (13.9%), and pyraclostrobin in 10 samples (13.9%) [22]. These findings are consistent with broader European monitoring data showing that multiple residues per sample are increasingly common in fruits and vegetables, particularly in intensively protected crops [8,18,19,23,24].

For the present risk assessment, the analysis focused on the active substances most frequently detected in apples and pears in 2022: captan, flonicamid, acetamiprid and fosetyl-Al in apples, and captan and acetamiprid in pears.

In apples, the mean residue levels ranged from 0.012 mg·kg^−1^ (acetamiprid) to 0.628 mg·kg^−1^ (fosetyl-Al), with corresponding P95 values of 0.043 and 3.088 mg·kg^−1^, respectively. In pears, the mean concentrations were 0.226 mg·kg^−1^ for captan and 0.010 mg·kg^−1^ for acetamiprid, with corresponding P95 values of 0.979 and 0.022 mg·kg^−1^.

All of these values remained well below the MRLs in force in 2022 (Table 5), which is consistent with the overall high level of regulatory compliance observed in the national control program.

In 2022, MRL exceedances in pome fruits were rare. Among the pesticides included in the present assessment, only fosetyl-Al exceeded the applicable MRL in one apple sample (1/442 samples; 0.23%). No exceedances were recorded for captan, flonicamid or acetamiprid in apples, nor for captan or acetamiprid in pears. These results indicate a high level of compliance with regulatory limits in apples and pears available on the Polish market.

In addition to the active substances included in the present quantitative risk assessment, several other pesticides were detected in apple and pear samples in 2022, typically at low frequencies and/or low concentration levels. These substances were not incorporated into the exposure calculations due to their sporadic occurrence or limited quantitative relevance within the context of the present assessment. Nevertheless, their detection reflects the complexity of residue profiles in intensively managed pome fruits and is documented in the national monitoring dataset.

These concentration patterns reflect current plant protection practices in commercial orchards, where captan and fosetyl-Al are widely used fungicides, and acetamiprid and flonicamid are commonly applied insecticides/acaricides. The presence of multiple residues in single samples is expected in intensively managed orchards, where 20–30 applications of plant protection products (PPPs) per season are not unusual [8,9]. However, the toxicological relevance of such mixtures needs to be considered in relation to dietary exposure and health-based guidance values rather than occurrence alone.

### 2.2. Dietary Exposure to Pesticide Residues (%ADI)

To place the measured residue levels in a dietary context, estimated daily intake (EDI) values expressed as a percentage of the acceptable daily intake (%ADI) were derived using body weight- and age-specific consumption scenarios. These were based on FAO/WHO GEMS/Food cluster diets and national data, covering a range of population groups including German children, UK infants and toddlers, Polish and German general adult populations, and the GEMS/Food G08 cluster (Table 6). The resulting %ADI values for the assessed pesticides in apples and pears are presented in Table 7.

For apples, the highest chronic exposure, expressed as %ADI, was generally observed in German children (DE child), who have the highest body weight-adjusted consumption among the evaluated scenarios. Even in this group, mean exposure remained modest: for captan, flonicamid and acetamiprid, the mean %ADI values were 1.25%, 0.92% and 0.61%, respectively, while for fosetyl-Al the mean %ADI was 0.59%. At the upper end of the distribution (P95), the corresponding %ADI values were still below 6% for captan (5.17%) and below 3% for flonicamid, acetamiprid and fosetyl-Al (2.19%, 2.14% and 2.89%, respectively). For the adult populations (Polish and German general populations, GEMS/Food G08, UK adults), the mean %ADI values were typically below 0.5%, with the P95 values substantially below 2%.

For pears, the exposure levels were even lower. In the most conservative scenario (Dutch toddlers, NL small child), the mean %ADI values for captan and acetamiprid were 0.39% and 0.17%, respectively, with P95 values of 1.70% and 0.38%, respectively (Table 7). For other child and adult scenarios, the mean and P95%ADI values for both pesticides were generally below 0.1% and 0.5%, respectively.

### 2.3. Non-Carcinogenic Risk Characterization (EDI, HQ and HI)

To further characterize non-carcinogenic risk, the EDIs were expressed in absolute terms (mg·kg^−1^ bw·day^−1^) and transformed into hazard quotients (HQ = EDI/ADI) for each pesticide–fruit–scenario combination. In addition, the Hazard Index (HI) was calculated as the sum of HQ values for multiple substances within each fruit and scenario. Key results for Polish adults, UK toddlers, the German general population, and the GEMS/Food G08 cluster are summarized in Table 8.

For apples, among Polish adults, the EDI values for captan, flonicamid, acetamiprid, and fosetyl-Al were in the order of 10^−4^–10^−3^ mg·kg^−1^ bw·day^−1^ (Table 8). The corresponding HQs ranged from approximately 9.8 × 10^−4^ (acetamiprid) to 2.0 × 10^−3^ (captan), with fosetyl-Al and flonicamid in between. The cumulative HI for these four substances was approximately 5.8 × 10^−3^, i.e., less than 1% of the threshold value of 1. These findings suggest that, for Polish adults, the chronic non-carcinogenic risk associated with these residues in apples is negligible.

In children, higher exposure levels were observed, consistent with their greater consumption per kilogram of body weight. In the German child (DE child) scenario, the EDIs for captan, flonicamid, acetamiprid, and fosetyl-Al in apples were approximately one order of magnitude higher than in Polish adults, resulting in HQ values up to about 1.2 × 10^−2^ for captan and 9.0 × 10^−3^ for flonicamid (Table 8). The cumulative HI for apples in this group was around 3.5 × 10^−2^, still substantially below 1. For UK toddlers, HQ and HI values were intermediate between those estimated for German children and adults in the other scenarios, but again, all HQs were clearly below 0.1, and the HI remained below 0.01.

In apples for the German general population and GEMS/Food G08, the HQs for the individual substances were generally in the 10^−4^–10^−3^ range, and the HIs were approximately 6.9 × 10^−3^ and 3.4 × 10^−3^, respectively (Table 8). These findings further support the conclusion that chronic dietary exposure to these residues from apple consumption is not expected to pose non-carcinogenic health risks in adults.

For pears, both the EDIs and HQs were lower than for apples across all of the scenarios considered. In Polish adults, the HQs for captan and acetamiprid were 6.33 × 10^−4^ and 1.12 × 10^−4^, respectively, yielding a cumulative HI of approximately 7.5 × 10^−4^ (Table 8). In the German child and Dutch toddler scenarios, the HQ values for captan reached, at most, the order of 10^−3^, with acetamiprid remaining at 10^−4^, and the HIs staying below 9 × 10^−4^. In the adult scenarios (German general population, GEMS/Food G08), the HQs were below 3 × 10^−4^ for captan and below 1 × 10^−4^ for acetamiprid, resulting in very low HIs (≈1–2 × 10^−4^).

### 2.4. Acute Exposure Assessment

An acute exposure screening was conducted for fosetyl-Al, the only pesticide that exceeded the applicable maximum residue limit (MRL) in apples in 2022 (1/89 samples; 1.12%).

The assessment was performed using the highest detected residue (HR = 2.40 mg·kg^−1^) and the most conservative consumption scenario (German child diet), representing the highest body weight-adjusted intake among the evaluated population groups.

Acute exposure was estimated using a deterministic worst-case approach according to the following equation:

Acute exposure = (HR × daily consumption)/body weight.

Under these conservative assumptions, the calculated acute exposure was: 0.030 mg × kg^−1^ body weight.

This calculation assumes that the entire daily fruit intake originates from a single lot containing the highest detected residue level and does not consider potential residue reduction due to washing, peeling, or processing. Therefore, the estimate represents a highly conservative exposure scenario.

The resulting value remains substantially below the acceptable daily intake (ADI = 1 mg·kg^−1^ bw·day^−1^). Furthermore, no acute reference dose (ARfD) has been established for fosetyl-Al by EFSA, indicating that no acute toxicological threshold has been identified for this substance.

For the remaining pesticides included in the present assessment (captan, flonicamid, and acetamiprid), no MRL exceedances were observed in apples or pears in 2022; consequently, no acute exposure scenario of regulatory concern was identified.

## 3. Discussion

The results presented herein indicate that all of the HQ values were far below 1 in every scenario, fruit, and pesticide considered. The cumulative HIs remained at least one order of magnitude below 0.1, even in high-consumption scenarios for children. Apples contributed more to the overall exposure and HI than pears, reflecting higher consumption and a broader range of active substances detected. Children consistently showed higher HQ and HI values than adults, which is expected given their higher intake per kilogram of body weight and emphasizes the relevance of child-specific exposure assessment.

From a risk assessment perspective, an HI below 1 is commonly interpreted as indicating that adverse non-cancer health effects are unlikely for the mixture under the exposure conditions considered [16,17,18]. The very low HI values obtained in this study (≤3.5 × 10^−2^ for apples and ≤9 × 10^−4^ for pears in the most conservative scenarios) therefore strongly suggest that current levels of pesticide residues in pome fruits on the Polish market are compatible with existing health-based guidance values.

It should be highlighted that the HIs presented here serve a screening purpose, as they were calculated by a simple summation of HQs across substances, without grouping by common mode of action. This approach tends to overestimate potential risks, because it assumes dose additivity across all substances, even when their toxicological profiles differ. According to cumulative risk assessment principles, more refined assessments should aggregate HQs only for substances sharing a common toxicological endpoint or mechanism (e.g., acetylcholinesterase inhibition) [16]. The low HIs observed in this screening context provide additional reassurance that more refined grouping would be unlikely to change the overall risk conclusions.

The present findings are broadly consistent with previous European and international assessments of dietary exposure to pesticide residues in fruits. Large-scale evaluations conducted by EFSA and national agencies have repeatedly shown that, while residues are frequently detected in fruits and vegetables, chronic dietary exposure typically remains well below health-based guidance values for both adults and children [8,13,19,25,26]. Similar conclusions have been reported in commodity-specific studies on fruit consumption, where the calculated HQ and HI values for pesticide mixtures are usually far below 1, even in high-consumption groups [17,18,19,24,27,28,29,30].

Our analysis adds to this body of evidence by providing a focused assessment for apples and pears, using recent monitoring data from Poland combined with harmonized GEMS/Food consumption scenarios. The data indicate that, under the current use patterns and residue levels, the chronic non-carcinogenic risk from pesticide residues in pome fruits is negligible for both children and adults. At the same time, the consistently higher HQ and HI values in children underscore the need to maintain age-stratified exposure assessment and to give particular attention to high-consumption subgroups.

The results also have practical implications for risk management and communication. First, they support the conclusion that the existing regulatory measures, including MRLs and authorization procedures, are effective in keeping dietary exposure within safe limits for the pesticides considered. Second, they underline the value of continued national monitoring, particularly for commodities with high consumption and intensive plant protection, such as apples. The monitoring of trends in multiple residues per sample and in %ADI or HI over time can help identify emerging issues and inform targeted interventions. Third, from a consumer perspective, the data suggest that promoting fruit consumption in line with dietary guidelines remains compatible with pesticide risk management, provided that official control systems continue to function effectively. Simple household practices, such as washing and peeling, though not explicitly modeled in this study, are likely to further reduce actual exposure to residues.

Finally, although this analysis focused on a selected set of active substances and scenarios, the methodology applied here—combining national monitoring data with standardized consumption models and calculating EDI, HQ and HI—can be readily extended to other pesticide–commodity combinations. Such applications would contribute to a more comprehensive understanding of dietary exposure profiles and support evidence-based public health recommendations on fruit consumption and pesticide risk mitigation.

## 4. Materials and Methods

### 4.1. Study Design and Data Sources

This study was designed as a deterministic dietary exposure and health-risk assessment using monitoring and consumption data. The analysis focused on apples and pears available on the Polish market and was conducted from the perspective of the Polish population, while also incorporating harmonized European consumption scenarios.

The data on pesticide residues were obtained from the official Polish monitoring and control program for pesticide residues in food for the year 2022, as compiled and interpreted by the NIZP PZH-PIB [22]. The monitoring covered a large number of active substances in apple and pear samples collected within the national control plan. For the present assessment, we selected the most frequently detected pesticides in pome fruits: captan, flonicamid, acetamiprid, and fosetyl-Al in apples, and captan and acetamiprid in pears. For each active substance, the mean concentrations and 95th percentile (P95) concentrations in the fruit samples were extracted (Table 5) [22].

The fruit consumption data were obtained from two sources: (i) Polish national statistics, including household budget surveys and agricultural market analyses, which provide information on the average annual and monthly consumption of apples in Polish households [10,22,23]; and (ii) the FAO/WHO GEMS/Food cluster diets, which supply harmonized body weight-specific fruit consumption values (g·kg^−1^ body weight·day^−1^) for different age groups and countries [22,24]. Because detailed national data on pear consumption were limited, pear intake in the risk assessment was primarily based on GEMS/Food and other European dietary scenarios.

The risk assessment was conducted separately for children and adults. The following representative body weights (BWs) and scenarios were considered: Polish general adult population (BW = 62.8 kg), German child (DE child), German general population (DE general), and GEMS/Food G08 cluster diet (children and adults), as shown in Table 6.

### 4.2. Fruit Consumption Scenarios

The average daily fruit consumption (Con, g·person^−1^·day^−1^) for apples and pears was derived from the sources described above and then converted to body weight-normalized intake (kg fruit·kg^−1^ bw·day^−1^) by dividing by the assumed body weight for each scenario [10]. For apples, the Polish national data indicated [22] an average annual per capita consumption of 10.68 kg in 2023, corresponding to approximately 29 g·person^−1^·day^−1^, while Central Statistical Office (GUS) [23] household budget surveys provided more detailed information by socio-economic group (Table 9 and Table 10) [10,22,23].

For the risk calculations, we used the harmonized daily intake values from Table 6, which summarize the average daily consumption of apples and pears for several European diets (DE child, UK infant, UK toddler, Polish general adult, UK adult, UK vegetarian adult, GEMS/Food G08, DE general adult, DE women 14–50 years).

For pears, the consumption data were taken primarily from GEMS/Food and European cluster diets, with scenarios including DE child, UK infant, UK toddler, NL toddler, PL general adult, UK adult, UK vegetarian adult, GEMS/Food G08, DE general adult, and DE women 14–50 years (Table 6). These scenarios allow for a realistic and conservative characterization of exposure in both Polish consumers and other European subpopulations, particularly children, who generally exhibit a higher intake per kilogram of body weight.

All of the consumption values used in the calculations are presented in Table 6 as both g·kg^−1^ bw·day^−1^ and g·person^−1^·day^−1^, allowing direct comparison across age groups and countries.

### 4.3. Pesticide Residue Data and Health-Risk Assessment

Based on the results of studies conducted in Poland by the Chief Sanitary Inspectorate (GIS) as part of official control, the National Institute of Public Health—National Institute of Hygiene prepared a report assessing the potential health risk to consumers resulting from the presence of pesticide residues in food available on the Polish market in 2022. According to this report, a total of 284 analyses for the presence of pesticides in citrus fruits and bananas were carried out under the official control program in 2022 (Table 5) [28]. The average concentrations of pesticides in citrus fruits and bananas available on the domestic market, as presented in the report, are shown in Table 6 [28]. Health risks associated with the consumption of pesticide residues in the analyzed citrus fruits and bananas were determined based on the following indicators [24,26,29,30]:

The mean and 95th percentile concentrations of the selected pesticides in apples and pears (C, mg·kg^−1^ fresh weight) were taken from the 2022 Polish national report on pesticide residues in food [22]. The selected compounds and their concentration statistics are summarized in Table 5.

For apples, the mean and P95 concentrations (mg·kg^−1^) were as follows: captan 0.249 and 1.036, flonicamid 0.018 and 0.044, acetamiprid 0.012 and 0.043, fosetyl-Al 0.628 and 3.088. For pears, the mean and P95 concentrations were 0.226 and 0.979 for captan, and 0.010 and 0.022 for acetamiprid. The corresponding MRL values valid in 2022 were also recorded (Table 5), but were used only as a regulatory reference, not as toxicological thresholds.

The estimated daily intake (EDI) of each pesticide (mg·kg^−1^ bw·day^−1^) from apples or pears was calculated using the standard equation:

EDI = *C* × C_on_/BW (1)

where

- *C* is the average concentration of the pesticide in the fruit (mg·kg^−1^),
- C_on_ is the average daily fruit consumption (kg·person^−1^·day^−1^),
- BW is body weight (kg) for the given population scenario.

For some comparisons with the Polish report, EDI values were also expressed as a percentage of the acceptable daily intake (%ADI), as presented in Table 7 [22].

Non-carcinogenic risk for each pesticide–fruit–scenario combination was characterized by the hazard quotient (HQ):

HQ = EDI/ADI(2)

where EDI is daily fruit consumption and ADI (mg·kg^−1^ bw·day^−1^) is the health-based guidance value established by EFSA and reported in the Polish monitoring report [22]. HQ values < 1 indicate that chronic dietary exposure does not exceed the ADI and is therefore not expected to pose a relevant non-cancer health concern.

To account for the potential combined effects of multiple residues in the same fruit, the cumulative Hazard Index (HI) was calculated as

(3) $$HI=\sum_{i=1}^{n}HQ_{i}$$

where HQ_i_ is the hazard quotient for the *i*-th pesticide in a given fruit and scenario. The HI was calculated separately for apples and pears and separately for each population group (children vs. adults and each diet scenario). In line with current practice, a HI < 1 was interpreted as indicating that the combined non-carcinogenic risk from the mixture of assessed pesticides is unlikely to be of concern, recognizing that this is a screening-level indicator that assumes dose addition across substances.

All calculations were performed deterministically using the mean concentration values as the primary exposure metric, while the P95 values were used to explore higher-end exposure under conservative assumptions. In addition to the chronic exposure assessment, the highest residue (HR) values presented in Table 5 were reviewed in the context of potential acute exposure. Acute risk was considered for substances for which MRL exceedances were observed. In the case of fosetyl-Al, which exceeded the MRL in one apple sample, an acute reference dose (ARfD) has not been established as necessary in recent EFSA evaluations; therefore, a classical ARfD-based acute risk characterization was not required. For the remaining substances, no MRL exceedances were recorded in apples or pears in 2022, and acute exposure was not expected to be of regulatory concern.

## 5. Conclusions

This quantitative risk assessment, utilizing 2022 Polish monitoring data and harmonized European consumption scenarios, indicates that chronic dietary exposure to the most frequently detected pesticide residues in apples and pears does not pose a relevant non-carcinogenic health concern for the assessed populations. For all scenarios, the hazard quotients (HQs) for individual pesticides and the cumulative Hazard Index (HI) remained significantly below the regulatory threshold of 1. The findings strongly suggest that current residue levels in the Polish market are well within safe limits. A key finding is that children (particularly in the German child and UK toddler scenarios) consistently demonstrated higher relative exposure (i.e., higher EDI and HQ values) than adults, which is directly attributable to their higher food consumption per kilogram of body weight. However, even in these worst-case pediatric scenarios, the cumulative HI remained well below 0.1, indicating a negligible risk. The results support the effectiveness of the current regulatory framework, including MRLs, in protecting consumer health. Furthermore, the findings confirm that public health recommendations promoting the consumption of pome fruits remain appropriate and are not compromised by the chronic health risks from the evaluated residues. The findings emphasize the importance of continued pesticide residue surveillance and routine age-specific dietary risk assessments for high-consumption fruits such as apples, to ensure that exposure levels remain within established health-based guidance values, particularly for children.

## Figures and Tables

**Table 1:** Cultivation area and production of apples and pears in Poland.

Cultivation Area (Thousand Ha)					
**Year**	**2020**	**2021**	**2022**	**2023**	**2024**
Total fruit	348.5	359.2	352.3	348.2	343.0
Including apples	152.6	161.9	151.9	150.0	148.0
Including pears	5.8	5.6	5.5	5.6	5.9
**Production—Harvest (Thousand tons)**					
Total fruit	4518.4	5059.5	5363.1	4924.4	4213.4
Including apples	3555.2	4067.4	4264.7	3892.7	3384.5
Including pears	61.0	68.6	80.6	79.0	74.2

**Table 2:** Apple and pear exports in 2020–2024 in Poland.

Exports (Thousand Tons)					
**Year**	**2020**	**2021**	**2022**	**2023**	**2024**
Total fruit	1001.5	1300.4	1065.5	1120.8	1060.0
Including apples	659.7	939.4	745.9	816.9	775.0
Including pears	99.1	120.2	108.1	109.3	99.0
**Season**	**2020/21**	**2021/22**	**2022/23**	**2023/24**	**2024/25**
Total fruit	1120.3	1168.7	1132.4	1096.3	1050.0
Including apples	767.3	845.7	801.6	803.5	760.0
Including pears	111.1	97.1	128.4	99.2	99.5

**Table 3:** Results of national official food control and monitoring for pesticide residues in 2017–2022 in Poland *.

Parameter	2017	2018	2019	2020	2021	2022
Total number of samples tested	2440	2555	2624	3246	3759	4925
Number of results ≥ LOQ **	3250	3708	3987	4424	5924	7741
Number of samples in which no pesticide residues were detected (%)	1245 (51.0%)	1202 (47.0%)	1194 (45.5%)	1503 (46.3%)	1613 (42.9%)	2198 (44.9%)
Number of samples containing at least one pesticide residue (%)	1113 (45.6%)	1254 (49.1%)	1430 (54.5%)	1559 (48.0%)	1923 (51.2%)	2375 (48.6%)
Number of samples in which residues of at least two pesticides were found (%)	680 (27.9%)	853 (33.4%)	896 (34.1%)	1073 (33.1%)	1462 (38.9%)	1831 (37.4%)
Number of results > MRL	106	135	159	208	279	374
Number of results not complying with MRL	53	70	73	127	157	160
Number of samples in which non-compliance(s) with MRL was found (%)	45 (1.8%)	52 (2.0%)	65 (2.5%)	112 (3.5%)	137 (3.6%)	144 (2.9%)
Number of pesticides found in at least 1 sample	147	148	167	146	192	187
Number of pesticides detected in at least 10 samples	61	66	70	73	80	99
Average number of results ≥ LOQ per sample	1.44	1.33	1.52	1.36	1.56	1.53
Number of compounds for which a long-term risk assessment was performed, Total	18	24	22	21	19	27

* National food control and monitoring for pesticide residues covered: vegetables, fruits, cereals and cereal products, oilseeds, animal products, processed products, products for infants and young children, and thickeners. ** LOQ—limit of quantification.

**Table 4:** Number of compounds and number of positive results in analyzed fruits covered by monitoring and official food control for pesticide residues in Poland in 2022.

Specification (Type of Fruit)	Number of Samples	Number of Pesticides Tested	Number of Pesticides Found	Average Number of Residues Per Sample	Number of Residues Per Sample	Number of Samples Exceeding MRL *n*, (%)
Apples	89	481	36	261	2.93	1 (1.12%)
Pears	72	479	29	159	2.21	0 (0.00%)

**Table 5:** Mean concentration, 95th percentile (P95), concentration range (min–max), highest residue (HR), and MRL values for the most frequently detected pesticides in apples and pears in Poland in 2022.

Pesticide	Mean (mg kg^−1^)	P95 (mg kg^−1^)	Range (mg kg^−1^)	Highest Residue (HR) (mg kg^−1^)	MRL 2022 (mg kg^−1^)	Samples > MRL (*n*)
			**Apples**			
Captan	0.249	1.036	<0.01–1.50	1.50	10	0
Flonicamid	0.018	0.044	<0.01–0.044 *	0.044	0.3	0
Acetamiprid	0.012	0.043	<0.01–0.043 *	0.043	0.4	0
Fosetyl-Al	0.628	3.088	<0.10–2.40	2.40	150 **	1
			**Pears**			
Captan	0.226	0.979	<0.01–1.10	1.10	10	0
Acetamiprid	0.010	0.022	<0.01–0.14	0.14	0.4	0

* For flonicamid and acetamiprid in apples, no higher values than those reflected in P95 were reported in the dataset. ** MRL refers to the sum of fosetyl, phosphonic acid and their salts, expressed as fosetyl.

**Table 6:** Average daily intake for selected pome fruits (critical diets are highlighted in bold italics).

**Apples**			
**Diet**	Average body weight * [kg]	**Daily intake** **[g kg^−1^ bw day^−1^]**	**Daily intake** **[g person^−1^ day^−1^]**
Children			
***DE child***	***16.15***	***12.4800***	***201.5520***
UK infant	8.70	1.5632	13.6000
UK toddler	14.60	1.7055	24.9000
Adults			
PL general	62.80	2.0430	128.3000
UK adult	76.00	0.4105	31.2000
UK adult vegetarian	66.70	0.5922	39.5000
GEMS/Food G08	60.00	1.2135	72.8100
DE general	76.37	2.4262	185.2860
DE women aged 14–50	67.47	2.5763	173.8252
**Pears**			
**Diet**	**Average body weight [kg]**	**Daily intake** **[g kg^−1^ bw day^−1^]**	**Daily intake** **[g person^−1^ day^−1^]**
Children			
DE child	16.15	0.6500	10.4975
UK infant	8.70	0.2529	2.2000
UK toddler	14.60	0.1781	2.6000
***NL toddler***	***10.20***	***4.3390***	***44.2578***
Adults			
PL general	62.80	0.2803	17.6000
UK toddler	76.00	0.0592	4.5000
UK adult vegetarian	66.70	0.0765	5.1000
GEMS/Food G08	60.00	0.1407	8.4400
DE general	76.37	0.1222	9.3350
DE women aged 14–50	67.47	0.1429	9.6390

* b.w.—body weight.

**Table 7:** Estimated daily intake (EDI) of the analyzed pesticide consumed from stone fruits, expressed as %ADI (results for the critical diet are shown in bold italics).

**Pesticide**	**Apples**								
Captan ADI 0.25 mg kg^−1^ bw day^−1^ [22]	***DE child***	UK infant	UK toddler	PL general	UK adult	UK adult vegetarian	GEMS/Food G08	DE general	DE women aged 14–50
Average	***1.25%***	0.16%	0.17%	0.20%	0.04%	0.06%	0.12%	0.24%	0.26%
P95	***5.17%***	0.65%	0.71%	0.85%	0.17%	0.25%	0.50%	1.01%	1.07%
Flonicamid ADI 0.025 mg kg^−1^ bw day^−1^ [22]	***DE child***	UK infant	UK toddler	PL general	UK adult	UK adult vegetarian	GEMS/Food G08	DE general	DE women aged 14–50
Average	***0.92%***	0.12%	0.13%	0.15%	0.03%	0.04%	0.09%	0.18%	0.19%
P95	***2.19%***	0.27%	0.30%	0.36%	0.07%	0.10%	0.21%	0.43%	0.45%
Acetamiprid ADI 0.025 mg kg^−1^ bw day^−1^ [22]	***DE child***	UK infant	UK toddler	PL general	UK adult	UK adult vegetarian	GEMS/Food G08	DE general	DE women aged 14–50
Average	***0.61%***	0.08%	0.08%	0.10%	0.02%	0.03%	0.06%	0.12%	0.13%
P95	***2.14%***	0.27%	0.29%	0.35%	0.07%	0.10%	0.21%	0.42%	0.44%
Fosetyl-AL ADI 1 mg kg^−1^ bw day^−1^ [22]	***DE child***	UK infant	UK toddler	PL general	UK adult	UK adult vegetarian	GEMS/Food G08	DE general	DE women aged 14–50
Average	***0.59%***	0.07%	0.08%	0.10%	0.02%	0.03%	0.06%	0.11%	0.12%
P95	***2.89%***	0.36%	0.39%	0.47%	0.10%	0.14%	0.28%	0.56%	0.60%
**Pesticide**	**Pears**								
Captan ADI 0.25 mg kg^−1^ bw day^−1^ [22]	DE child	***NL toddler***	UK infant	PL general	UK adult	UK adult vegetarian	GEMS/Food G08	DE general	DE women aged 14–50
Average	0.06%	***0.39%***	0.02%	0.03%	0.01%	0.01%	0.01%	0.01%	0.01%
P95	0.25%	***1.70%***	0.10%	0.11%	0.02%	0.03%	0.06%	0.05%	0.06%
Acetamiprid ADI 0.025 mg kg^−1^ bw day ^−1^ [22]	DE child	***NL toddler***	UK infant	PL general	UK adult	UK adult vegetarian	GEMS/Food G08	DE general	DE women aged 14–50
Average	0.03%	***0.17%***	0.01%	0.01%	0.00%	0.00%	0.01%	0.00%	0.01%
P95	0.06%	***0.38%***	0.02%	0.02%	0.01%	0.01%	0.01%	0.01%	0.01%

**Table 8:** EDI, HQ, and HI values for residues of selected active substances in stone fruits consumed by PL adults (As), DE (Ch), DE (gen), UK (toddlers) and GEMS/Food G08.

Type of Pome Fruit	EDI				
	Pesticide Active Substance				
	PL (A)	DE (Ch)	DE (Gen)	UK (Toddlers)	GEMS/Food G08
Apples	Captan				
	5.09 × 10^−4^	3.11 × 10^−3^	6.04 × 10^−4^	4.25 × 10^−4^	3.02 × 10^−4^
	Flonicamid				
	3.68 × 10^−5^	2.25 × 10^−4^	4.37 × 10^−5^	3.07 × 10^−5^	2.19 × 10^−5^
	Acetamiprid				
	2.45 × 10^−5^	1.50 × 10^−4^	2.92 × 10^−5^	2.05 × 10^−5^	1.46 × 10^−5^
	Fosetyl-Al				
	1.28 × 10^−3^	7.84 × 10^−3^	1.52 × 10^−3^	1.07 × 10^−4^	7.32 × 10^−2^
	HQ				
	Captan				
	2.03 × 10^−3^	1.24 × 10^−2^	2.42 × 10^−3^	1.70 × 10^−3^	1.21 × 10^−3^
	Flonicamid				
	1.47 × 10^−3^	8.99 × 10^−3^	1.75 × 10^−3^	1.23 × 10^−4^	8.74 × 10^−4^
	Acetamiprid				
	9.81 × 10^−4^	6.00 × 10^−3^	1.17 × 10^−3^	8.19 × 10^−4^	5.82 × 10^−4^
	Fosetyl-Al				
	1.28 × 10^−3^	7.84 × 10^−3^	1.52 × 10^−3^	3.02 × 10^−4^	7.32 × 10^−4^
	HI				
	≈5.77 × 10^−3^	≈3.52 × 10^−2^	≈6.85 × 10^−3^	4.82 × 10^−3^	3.43 × 10^−3^
Pears	EDI				
	Captan				
	6.33 × 10^−5^	1.47 × 10^−4^	2.76 × 10^−5^	4.02 × 10^−5^	3.18 × 10^−5^
	Acetamiprid				
	2.80 × 10^−6^	6.51 × 10^−6^	1.22 × 10^−6^	1.78 × 10^−6^	1.41 × 10^−6^
	HQ				
	Captan				
	6.33 × 10^−4^	5.88 × 10^−4^	2.76 × 10^−4^	1.61 × 10^−4^	1.27 × 10^−4^
	Acetamiprid				
	1.12 × 10^−4^	2.60 × 10^−4^	4.89 × 10^−5^	7.12 × 10^−5^	5.63 × 10^−5^
	HI				
	≈3.65 × 10^−4^	≈8.48 × 10^−4^	≈1.60 × 10^−4^	≈2.32 × 10^−4^	1.84 × 10^−4^

**Table 9:** Average monthly apple consumption per person in households by socio-economic group in Poland in 2023.

Specification [in kg]	Grand Total	Households of Which							
		Employees			Farmers	Self-Employed	Retirees and Pensioners		
		Total	In				Total	Retirees	Pensioners
			Manual Labor Positions	Non-Manual Labor Positions					
Fruit [in kg]	3.71	3.45	3.02	3.81	2.61	3.83	4.69	4.70	4.51
Of which apples	0.89	0.80	0.74	0.84	0.65	0.84	1.24	1.25	1.18

**Table 10:** Average annual apple consumption in households in Poland (kg per person per year).

Specification	2019	2020	2021	2022	2023
Fresh and chilled fruit	42.84	43.44	44.28	41.28	41.76
Including apples	12.12	11.28	11.16	10.44	10.68

## Data Availability

Detailed data are available from the authors.

## Abbreviations

The following abbreviations are used in this manuscript:

ADI	Acceptable Daily Intake
ARfD	Acute Reference Dose
CRA	Cumulative Risk Assessment
CSF	Cancer Slope Factor
EDI	Estimated Daily Intake
EFSA	European Food Safety Authority
ELCR	Excess Lifetime Cancer Risk
GAP	Good Agricultural Practices
GIS	Chief Sanitary Inspectorate in Poland
GUS	Central Statistical Office in Poland
HI	Hazard Index
HQ	Hazard Quotient
LOQ	Limit of Quantification
MRL	Maximum Residue Level
PPP	Plant Protection Product
RASFF	Rapid Alert System for Food and Feed
US EPA	U.S. Environmental Protection Agency
WHO	World Health Organization
